# An assessment of the autism neuroimaging literature for the prospects of re-executability

**DOI:** 10.12688/f1000research.25306.2

**Published:** 2021-03-04

**Authors:** Steven M. Hodge, Christian Haselgrove, Leah Honor, David N. Kennedy, Jean A. Frazier

**Affiliations:** 1 Eunice Kennedy Shriver Center, Department of Psychiatry, University of Massachusetts Medical School, Worcester, Massachusetts, 01655, USA; 2 Lamar Soutter Library, University of Massachusetts Medical School, Worcester, Massachusetts, 01655, USA

**Keywords:** Reproducible Science, Neuroimaging, Autism, Data Sharing, Re-executability

## Abstract

**Background:** The degree of reproducibility of the neuroimaging literature in psychiatric application areas has been called into question and the issues that relate to this reproducibility are extremely complex. Some of these complexities have to do with the underlying biology of the disorders that we study and others arise due to the technology we apply to the analysis of the data we collect. Ultimately, the observations we make get communicated to the rest of the community through publications in the scientific literature.

**Methods:** We sought to perform a ‘re-executability survey’ to evaluate the recent neuroimaging literature with an eye toward seeing if the technical aspects of our publication practices are helping or hindering the overall quest for a more reproducible understanding of brain development and aging. The topic areas examined include availability of the data, the precision of the imaging method description and the reporting of the statistical analytic approach, and the availability of the complete results. We applied the survey to 50 publications in the autism neuroimaging literature that were published between September 16, 2017 to October 1, 2018.

**Results:** The results of the survey indicate that for the literature examined, data that is not already part of a public repository is rarely available, software tools are usually named but versions and operating system are not, it is expected that reasonably skilled analysts could approximately perform the analyses described, and the complete results of the studies are rarely available.

**Conclusions:** We have identified that there is ample room for improvement in research publication practices. We hope exposing these issues in the retrospective literature can provide guidance and motivation for improving this aspect of our reporting practices in the future.

## Introduction

There is concern about the status of reproducibility in science in general and neuroimaging neuroscience in particular ( Button *et al.*, 2013; Gorgolewski & Poldrack, 2016). A particularly germane concern was expressed by Kapur and colleagues in lamenting: “a profusion of statistically significant, but minimally differentiating, biological findings; ‘approximate replications’ of these findings in a way that neither confirms nor refutes them” ( Kapur *et al.*, 2012). The replication of a specific finding (or reproducibility of a specific analysis), as reflected in a publication, has many details and nuances to it ( Kennedy *et al.*, 2019). Often, we are searching for the ‘generalizability’ of a finding: does the finding hold true when using ‘similar’ data and a ‘similar’ analysis. The similarity of data (or analysis) is a fuzzy concept. One could have a population with the same number of subjects with the same diagnosis, having the same mean age and same gender distribution as a target population; however, if the diagnosis in question is a ‘spectrum’-diagnosis (for example, autism, schizophrenia, depression, etc.), despite the ‘sameness’ of my sample in the aforementioned categories, the detailed nature of the characteristics of my sample in the features of the diagnosis itself can still be quite variable. At the level of a biological finding, we typically do not predicate the finding on an exact acquisition protocol, or a specific analysis protocol; rather, it is implicit in our finding that it should hold for other valid acquisitions and analyses of the reported types. There is increasing evidence that this implicit assumption of similarity, when it relates to the specific details of acquisition or analysis, does not necessarily hold ( Glatard *et al.*, 2015).

**Figure 1.**
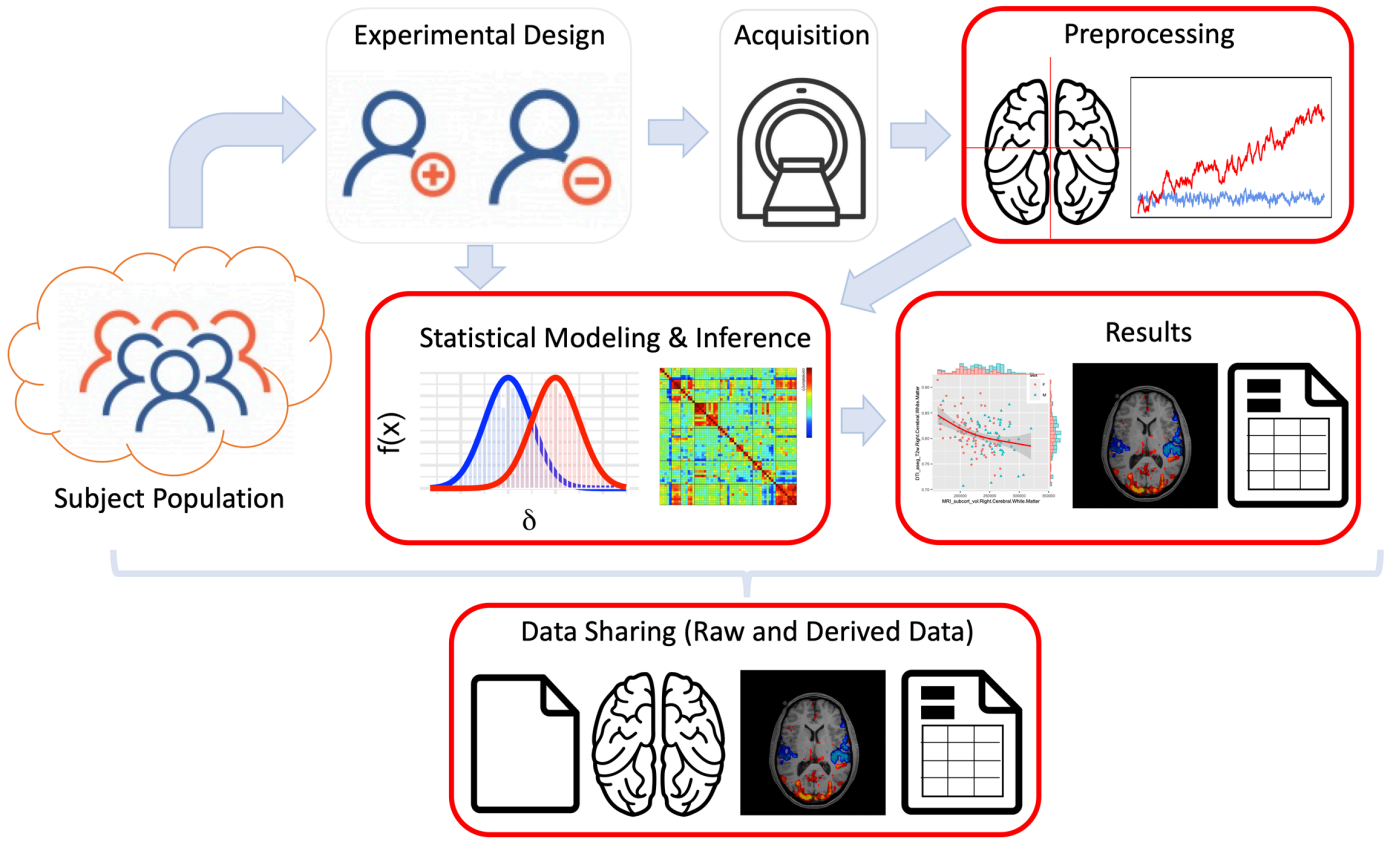
Essential elements of a publication. Elements of a publication that comprise a starting point for a structured exploration of the generalizability of a specific finding. The outlined areas define the technical prospects of re-executability of a finding that are evaluated in this survey.

Some have argued that the starting point for the structured exploration of the generalizability of a specific finding (and thus a cornerstone to the quest for reproducibility) lies in the original finding itself being re-executable ( Ghosh *et al.*, 2017; Kennedy, 2019). Starting from the re-execution of a finding will allow for the systematic exploration of the generalizability of that finding, over changes in data and analysis. To date, when new studies find different findings from prior studies, it is too easy to simply argue that differences in the subject population or analysis workflow differences account for the discrepancy. In this paper we concentrate on assessing the technical prospects of re-executability of a publication. As introduced above, there are many other factors that will contribute to the actual generalization of the findings including subject population details, data acquisition details, the nature of the processing and statistics (even if they can be re-executed), the underlying biological effect size, if present, etc. (see Figure 1). Take for example, the subject population. Too often researchers communicate a finding based on a convenience sample without any statement indicating that the results might not generalize to a sample that more accurately reflects human diversity (e.g. DeJesus *et al.*, 2019; Henrich *et al.*, 2010; Hruschka *et al.*, 2018; Rad *et al.*, 2018). Comprehensive and standardized description of all these additional factors are critical as well, but are beyond the scope of this evaluation. Our groups and others are looking into reporting standards for these areas as well.

The potential impact of reproducibility issues become most obvious when trying to make sense of the accumulated literature on specific topic areas ( Rane *et al.*, 2015). For this reason, we have chosen a particular area, ‘autism’ as a way to focus the literature for this survey, so that the conclusions we reach can have potential specific implications for that topic area. We feel that the autism focus, however, will generate findings that will have similar implications to other psychiatric and developmental application areas.

In this paper, we: 1) develop a specification for what constitutes an assessment of the technical re-executability for a given publication in each of the domains of: data, software, execution environment, statistics and results; 2) codify this assessment in survey form; and 3) apply the survey to a subset of the autism neuroimaging literature published recently (~2018). From the results of this survey, we can begin to generalize the state of the re-executability of the recent autism neuroimaging literature, in order to identify trends and opportunities for the enhancement of the re-executability status in support of greater overall generalizability (and hence reproducibility) of the literature. The survey template could also be applied as part of the publication review process, in order to prospectively attempt to enhance these aspects of reproducibility.

## Methods

### Survey development

Following the concept of a ‘re-executable publication’ ( Kennedy, 2019), in order to assess the prospects of re-execution of a given paper, we assess 1) the availability of the starting data, 2) the perceived completeness of the analysis description (both data processing and statistical assessment), and 3) the availability of the detailed complete results (in order to verify accuracy of re-execution). Regarding the ‘availability of the starting data’, we assess if the publication indicates how someone ^1^ (other than the authors themselves) could appropriately access the data. The ‘precision of the analysis description’ ultimately asks if a reader who is reasonably skilled in the necessary domains, could precisely carry out the prescribed analysis steps. Specifically, are the software versions, operating system and complete parameters somehow made available to the reader? The ‘detailed complete results’ assesses if the publication indicates how to obtain the complete results, in order to both verify that the re-execution generates the same result and to overcome the limitations of only a selected summary being presented, which impedes a more complete meta-analysis of the literature.

In each of the three assessment areas, the survey distinguished between the theoretical potential for reproduction (such as complete descriptions of data used, software and commands executed, and statistical tests applied) and the practical potential for reproduction (whether the data is in fact accessible, whether the software is still available and will run). While the survey did not require the raters to actually reproduce the various steps, they were asked to use their professional judgement and past experience to determine the potential reproducibility. In these ‘judgement’ questions we allow responses of ‘Yes’, ‘Approximately’, ‘I’m not sure’, and ‘No’ to allow some degree of confidence in these judgements. For ‘results availability’, we coded ‘Yes’ if all of the results were indicated as being available, ‘Partially’ if some of the results were indicated as being available, and ‘No’ if none of the results were indicated as available or no indication of the results availability was provided. Note that our assessments are not if the analysis or data accessibility is ‘optimum’, or even ‘correct’, but rather if the assessor could redo the approach as described.

**Figure 2.**
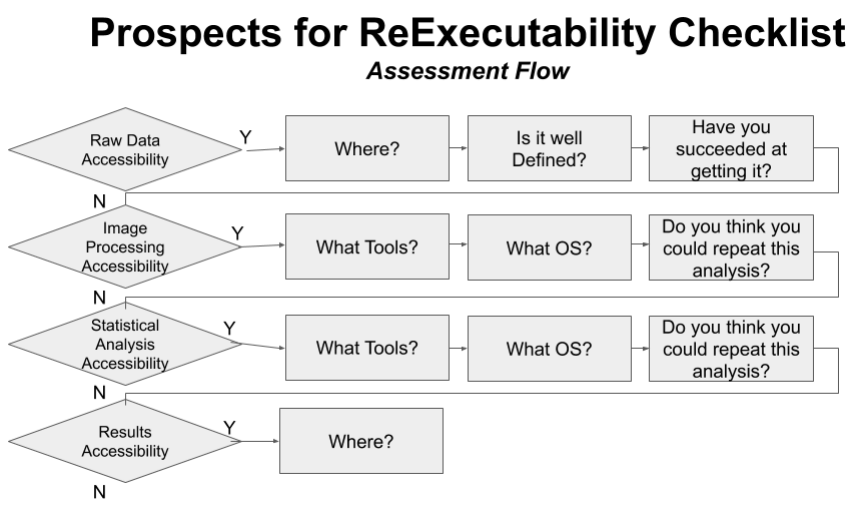
High-level survey design. OS, operating system.

Figure 2 provides an overview of the survey design.

The survey was constructed in Google Forms. The details of the logic and wording of the survey forms was piloted (10 articles, three raters) within our own group, and then released for public comment to the BrainHack Slack ^2^ channel in August, 2018. The final complete (serialized) text of the survey is provided in S1 (see *Extended data*; Hodge *et al.*, 2020c).

### Literature identification

On January 23, 2019, the following PubMed query was executed:

(("autistic disorder"[MeSH Terms] OR ("autistic"[All Fields] AND "disorder"[All Fields]) OR "autistic disorder"[All Fields] OR "autism"[All Fields]) AND ("magnetic resonance imaging"[MeSH Terms] OR ("magnetic"[All Fields] AND "resonance"[All Fields] AND "imaging"[All Fields]) OR "magnetic resonance imaging"[All Fields] OR "mri"[All Fields])) AND ("2014/01/25"[PDat] : "2019/01/23"[PDat] AND "humans"[MeSH Terms])

This is the expansion of the general query for ‘autism AND MRI, qualified to select publications between 1/25/2014 - 1/23/2019 and where the MeSH term includes ‘human’. This query generated 811 resultant publications at the time of the query (see S2, *Underlying data;* Hodge *et al.*, 2020a). We note that re-running the query today will generate additional results due to publications that have been added to PubMed after the search date but with publication dates within the defined range.

### Survey application

Starting from the most recent publication and working backwards, we reviewed the title and abstract to verify publications that were indeed neuroimaging studies (not a case report or review), in English, related to autism and for which we could access the full text of the article. Working backwards from publication date, we selected the first 50 publications that met the above criteria. Of these 50 publications, 38 were available as free full text on PubMed, three were available as a PDF through a general Google Scholar search (publisher/author provided), two were available in PDF format from ResearchGate, and seven did not seem to be available without institutional access. The survey was applied to each paper by one of three raters (DNK, SMH, CH). Each of the final results were reviewed by a second rater (DNK or SMH) and consensus reached with the original rater if discrepancies were found.

## Results

### Literature selected

The final set of publications used in this report is tabulated in Table 1. The publication dates span from September 16, 2017 to October 1, 2018. Publications from 27 different journals are included. The publications selected covered a number of different MRI-based techniques (structural N=20, task-based fMRI N=14, resting-state fMRI N=13, diffusion MRI N=11 and magnetic resonance spectroscopy N=5) ^3^. In this table we indicate what ‘type’of imaging was performed: structural MRI (S), task-based functional MRI (F), resting state fMRI (RS), diffusion MRI (D), magnetic resonance spectroscopy (MRS), arterial spin labeling (ASL).

**Table 1.** Publications Included in the survey.

First Author	Title	Reference	PMID	Imaging Type	Assessors (Primary/ Check)
Marusak HA ( Marusak *et al.*, 2018)	Mindfulness and dynamic functional neural connectivity in children and adolescents.	Behav Brain Res. 2018 Jan 15;336:211-218. doi: 10.1016/ j.bbr.2017.09.010. Epub 2017 Sep 5.	28887198	RS	Rev1/Rev3
Ramot M ( Ramot *et al.*, 2017)	Direct modulation of aberrant brain network connectivity through real-time NeuroFeedback.	Elife. 2017 Sep 16;6. pii: e28974. doi: 10.7554/eLife.28974.	28917059	F	Rev2/Rev3
Bruno JL ( Bruno *et al.*, 2017)	Longitudinal identification of clinically distinct neurophenotypes in young children with fragile X syndrome.	Proc Natl Acad Sci U S A. 2017 Oct 3;114(40):10767-10772. doi: 10.1073/ pnas.1620994114. Epub 2017 Sep 18.	28923933	S	Rev2/Rev3
Bottelier MA ( Bottelier *et al.*, 2017)	Age-dependent effects of acute methylphenidate on amygdala reactivity in stimulant treatment-naive patients with Attention Deficit/Hyperactivity Disorder.	Psychiatry Res Neuroimaging. 2017 Nov 30;269:36-42. doi: 10.1016/j.pscychresns.20 17.09.009. Epub 2017 Sep 12.	28938219	F	Rev2/Rev3
Hotier S ( Hotier *et al.*, 2017)	Social cognition in autism is associated with the neurodevelopment of the posterior superior temporal sulcus.	Acta Psychiatr Scand. 2017 Nov;136(5):517- 525. doi: 10.1111/acps.12814. Epub 2017 Sep 22.	28940401	S	Rev2/Rev3
Chien YL ( Chien *et al.*, 2017)	Altered white-matter integrity in unaffected siblings of probands with autism spectrum disorders.	Hum Brain Mapp. 2017 Dec;38(12):6053- 6067. doi: 10.1002/ hbm.23810. Epub 2017 Sep 20.	28940697	D	Rev2/Rev3
Braden BB ( Braden *et al.*, 2017)	Executive function and functional and structural brain differences in middle-age adults with autism spectrum disorder.	Autism Res. 2017 Dec;10(12):1945-1959. doi: 10.1002/ aur.1842. Epub 2017 Sep 21.	28940848	S, F	Rev3/Rev1
Hegarty JP 2nd ( Hegarty *et al.*, 2018)	A proton MR spectroscopy study of the thalamus in twins with autism spectrum disorder.	Prog Neuropsychopharmacol Biol Psychiatry. 2018 Feb 2;81:153-160. doi: 10.1016/ j.pnpbp.2017.09.016. Epub 2017 Sep 21.	28941767	MRS	Rev3/Rev1
Joshi G ( Joshi *et al.*, 2017)	Integration and Segregation of Default Mode Network Resting-State Functional Connectivity in Transition-Age Males with High-Functioning Autism Spectrum Disorder: A Proof-of- Concept Study.	Brain Connect. 2017 Nov;7(9):558-573. doi: 10.1089/ brain.2016.0483.	28942672	RS	Rev1/Rev3
Carlisi CO ( Carlisi *et al.*, 2017)	Shared and Disorder-Specific Neurocomputational Mechanisms of Decision-Making in Autism Spectrum Disorder and Obsessive-Compulsive Disorder.	Cereb Cortex. 2017 Dec 1;27(12):5804-5816. doi: 10.1093/ cercor/bhx265.	29045575	F	Rev1/Rev3
White T ( White *et al.*, 2018b)	Paediatric population neuroimaging and the Generation R Study: the second wave.	Eur J Epidemiol. 2018 Jan;33(1):99-125. doi: 10.1007/s10654- 017-0319-y. Epub 2017 Oct 24.	29064008	S, RS, D	Rev1/Rev3
Zhang F ( Zhang *et al.*, 2018)	Whole brain white matter connectivity analysis using machine learning: An application to autism.	Neuroimage. 2018 May 15;172:826-837. doi: 10.1016/j.neur oimage.2017.10.029. Epub 2017 Oct 25.	29079524	D	Rev1/Rev3
Stanfield AC ( Stanfield *et al.*, 2017)	Dissociation of Brain Activation in Autism and Schizotypal Personality Disorder During Social Judgments.	Schizophr Bull. 2017 Oct 21;43(6):1220-1228. doi: 10.1093/ schbul/sbx083.	29088456	F	Rev3/Rev1
Ni HC ( Ni *et al.*, 2018)	Neural correlates of impaired self-regulation in male youths with autism spectrum disorder: A voxel-based morphometry study.	Prog Neuropsychopharmacol Biol Psychiatry. 2018 Mar 2;82:233-241. doi: 10.1016/ j.pnpbp.2017.11.008. Epub 2017 Nov 9.	29129723	S	Rev3/Rev1
Murakami Y ( Murakami *et al.*, 2018)	Autistic traits modulate the activity of the ventromedial prefrontal cortex in response to female faces.	Neurosci Res. 2018 Aug;133:28-37. doi: 10.1016/ j.neures.2017.11.003. Epub 2017 Nov 12.	29141188	F	Rev3/Rev1
Balci TB ( Balci *et al.*, 2018)	Broad spectrum of neuropsychiatric phenotypes associated with white matter disease in PTEN hamartoma tumor syndrome.	Am J Med Genet B Neuropsychiatr Genet. 2018 Jan;177(1):101-109. doi: 10.1002/ajmg. b.32610. Epub 2017 Nov 20.	29152901	S	Rev3/Rev1
Naaijen J ( Naaijen *et al.*, 2018)	Striatal structure and its association with N-Acetylaspartate and glutamate in autism spectrum disorder and obsessive compulsive disorder.	Eur Neuropsychopharmacol. 2018 Jan;28(1):118-129. doi: 10.1016/ j.euroneuro.2017.11.010. Epub 2017 Nov 21.	29169826	S, MRS	Rev1/Rev3
Abbott AE ( Abbott *et al.*, 2018)	Repetitive behaviors in autism are linked to imbalance of corticostriatal connectivity: a functional connectivity MRI study.	Soc Cogn Affect Neurosci. 2018 Jan 1;13(1):32-42. doi: 10.1093/scan/nsx129.	29177509	RS	Rev3/Rev1
White T ( White *et al.*, 2018a)	Automated quality assessment of structural magnetic resonance images in children: Comparison with visual inspection and surface-based reconstruction.	Hum Brain Mapp. 2018 Mar;39(3):1218- 1231. doi: 10.1002/ hbm.23911. Epub 2017 Dec 5.	29206318	S	Rev3/Rev1
Forde NJ	Multi-modal imaging investigation of anterior cingulate cortex cytoarchitecture in neurodevelopment	Eur Neuropsychopharmacol. 2018 Jan; 28(1):13-23. doi: 10.1016/ j.euroneuro.2017.11.021. Epub 2017 Dec 7.	29223496	S, D, MRS	Rev1/Rev3
Wei L ( Wei *et al.*, 2018)	Aberrant development of the asymmetry between hemispheric brain white matter networks in autism spectrum disorder.	Eur Neuropsychopharmacol. 2018 Jan; 28(1):48-62. doi: 10.1016/ j.euroneuro.2017.11.018. Epub 2017 Dec 7.	29224969	D, S	Rev1/Rev3
Wadsworth HM ( Wadsworth *et al.*, 2018)	Action simulation and mirroring in children with autism spectrum disorders.	Behav Brain Res. 2018 Apr 2;341:1-8. doi: 10.1016/ j.bbr.2017.12.012. Epub 2017 Dec 13.	29247748	F	Rev1/Rev3
Bernas A ( Bernas *et al.*, 2018)	Wavelet coherence-based classifier: A resting-state functional MRI study on neurodynamics in adolescents with high- functioning autism.	Comput Methods Programs Biomed. 2018 Feb;154:143-151. doi: 10.1016/ j.cmpb.2017.11.017. Epub 2017 Nov 16.	29249338	RS	Rev3/Rev1
Alexander LM ( Alexander *et al.*, 2017)	An open resource for transdiagnostic research in pediatric mental health and learning disorders.	Sci Data. 2017 Dec 19;4:170181. doi: 10.1038/ sdata.2017.181.	29257126	RS, S, D	Rev3/Rev1
Gibbard CR ( Gibbard *et al.*, 2018)	Structural connectivity of the amygdala in young adults with autism spectrum disorder.	Hum Brain Mapp. 2018 Mar;39(3):1270- 1282. doi: 10.1002/ hbm.23915. Epub 2017 Dec 19.	29265723	S, D	Rev3/Rev1
Dona O ( Dona *et al.*, 2017)	Temporal fractal analysis of the rs-BOLD signal identifies brain abnormalities in autism spectrum disorder.	PLoS One. 2017 Dec 22;12(12):e0190081. doi: 10.1371/ journal.pone.0190081. eCollection 2017.	29272297	RS	Rev1/Rev3
Feczko E ( Feczko *et al.*, 2018)	Subtyping cognitive profiles in Autism Spectrum Disorder using a Functional Random Forest algorithm.	Neuroimage. 2018 May 15;172:674-688. doi: 10.1016/ j.neur oimage.2017.12.044. Epub 2017 Dec 21.	29274502	F	Rev3/Rev1
Ciaramidaro A ( Ciaramidaro *et al.*, 2018)	Transdiagnostic deviant facial recognition for implicit negative emotion in autism and schizophrenia.	Eur Neuropsychopharmacol. 2018 Feb;28(2):264-275. doi: 10.1016/ j.euroneuro.2017.12.005. Epub 2017 Dec 21.	29275843	F	Rev3/Rev1
Ktena SI ( Ktena *et al.*, 2018)	Metric learning with spectral graph convolutions on brain connectivity networks.	Neuroimage. 2018 Apr 1;169:431-442. doi: 10.1016/j.neuroi mage.2017.12.052. Epub 2017 Dec 24.	29278772	RS	Rev3/Rev1
Hu Y ( Hu *et al.*, 2018)	The neural substrates of procrastination: A voxel-based morphometry study.	Brain Cogn. 2018 Mar;121:11-16. doi: 10.1016/ j.bandc.2018.01.001. Epub 2018 Jan 6.	29309854	S	Rev2/Rev3
Kohls G ( Kohls *et al.*, 2018)	Altered reward system reactivity for personalized circumscribed interests in autism.	Mol Autism. 2018 Jan 30;9:9. doi: 10.1186/ s13229-018-0195- 7. eCollection 2018.	29423135	F	Rev3/Rev1
Boets B ( Boets *et al.*, 2018)	Alterations in the inferior longitudinal fasciculus in autism and associations with visual processing: a diffusion- weighted MRI study.	Mol Autism. 2018 Feb 8;9:10. doi: 10.1186/ s13229-018- 0188-6. eCollection 2018.	29449909	D	Rev3/Rev1
Stivaros S ( Stivaros *et al.*, 2018)	Randomised controlled trial of simvastatin treatment for autism in young children with neurofibromatosis type 1 (SANTA).	Mol Autism. 2018 Feb 22;9:12. doi: 10.1186/ s13229-018- 0190-z. eCollection 2018.	29484149	MRS, ASL, S, RS	Rev3/Rev1
Floris DL ( Floris *et al.*, 2018)	Network-specific sex differentiation of intrinsic brain function in males with autism.	Mol Autism. 2018 Mar 6;9:17. doi: 10.1186/ s13229-018- 0192-x. eCollection 2018.	29541439	RS	Rev3/Rev1
Adamson K ( Adamson & Troiani 2018)	Distinct and overlapping fusiform activation to faces and food.	Neuroimage. 2018 Jul 1;174:393-406. doi: 10.1016/j.neuroim age.2018.02.064. Epub 2018 Mar 22.	29578027	F	Rev3/Rev1
Li SJ ( Li *et al.*, 2018)	Alterations of White Matter Connectivity in Preschool Children with Autism Spectrum Disorder.	Radiology. 2018 Jul;288(1):209-217. doi: 10.1148/ radiol.2018170059. Epub 2018 Mar 27.	29584599	D	Rev3/Rev1
Sen B ( Sen *et al.*, 2018)	A general prediction model for the detection of ADHD and Autism using structural and functional MRI.	PLoS One. 2018 Apr 17;13(4):e0194856. doi: 10.1371/ journal.pone.0194856. eCollection 2018.	29664902	S, RS	Rev1/Rev3
Tsoi L ( Tsoi *et al.*, 2018)	Neural substrates for moral judgments of psychological versus physical harm.	Soc Cogn Affect Neurosci. 2018 May 1;13(5):460-470. doi: 10.1093/scan/nsy029.	29718384	F	Rev1/Rev3
Guzman GEC ( Guzman *et al.*, 2018)	Identification of alterations associated with age in the clustering structure of functional brain networks.	PLoS One. 2018 May 24;13(5):e0195906. doi: 10.1371/ journal.pone.0195906. eCollection 2018.	29795565	RS	Rev1/Rev3
Karahanoƒülu FI ( Karahanoğlu *et al.*, 2018)	Diffusion-weighted imaging evidence of altered white matter development from late childhood to early adulthood in Autism Spectrum Disorder.	Neuroimage Clin. 2018 Jun 7;19:840-847. doi: 10.1016/ j.nicl.2018.06.002. eCollection 2018.	29946509	D	Rev3/Rev1
Zhao G ( Zhao *et al.*, 2018)	Reduced structural complexity of the right cerebellar cortex in male children with autism spectrum disorder.	PLoS One. 2018 Jul 11;13(7):e0196964. doi: 10.1371/journal. pone.0196964. eCollection 2018.	29995885	S	Rev3/Rev1
Yan W ( Yan *et al.*, 2018)	Aberrant hemodynamic responses in autism: Implications for resting state fMRI functional connectivity studies.	Neuroimage Clin. 2018 Apr 13;19:320-330. doi: 10.1016/ j.nicl.2018.04.013. eCollection 2018.	30013915	RS	Rev3/Rev1
Kim N ( Kim *et al.*, 2018)	Aberrant Neural Activation Underlying Idiom Comprehension in Korean Children with High Functioning Autism Spectrum Disorder.	Yonsei Med J. 2018 Sep;59(7):897-903. doi: 10.3349/ ymj.2018.59.7.897.	30091324	F	Rev3/Rev1
Duret P ( Duret *et al.*, 2018)	Gyrification changes are related to cognitive strengths in autism.	Neuroimage Clin. 2018 Aug 4;20:415-423. doi: 10.1016/ j.nicl.2018.04.036. eCollection 2018.	30128280	S	Rev3/Rev1
Na S ( Na *et al.*, 2018)	White matter network topology relates to cognitive flexibility and cumulative neurological risk in adult survivors of pediatric brain tumors.	Neuroimage Clin. 2018 Aug 10;20:485-497. doi: 10.1016/ j.nicl.2018.08.015. eCollection 2018.	30148064	D	Rev3/Rev1
Chin R ( Chin *et al.*, 2018)	Recognition of Schizophrenia with Regularized Support Vector Machine and Sequential Region of Interest Selection using Structural Magnetic Resonance Imaging.	Sci Rep. 2018 Sep 14;8(1):13858. doi: 10.1038/s41598-018- 32290-9.	30218016	S	Rev2/Rev3
Gertsvolf N ( Gertsvolf *et al.*, 2018)	Association between Subcortical Morphology and Cerebral White Matter Energy Metabolism in Neonates with Congenital Heart Disease.	Sci Rep. 2018 Sep 19;8(1):14057. doi: 10.1038/s41598-018- 32288-3.	30232359	S, MRS	Rev2/Rev3
Gray JC ( Gray *et al.*, 2018)	No evidence for morphometric associations of the amygdala and hippocampus with the five-factor model personality traits in relatively healthy young adults.	PLoS One. 2018 Sep 20;13(9):e0204011. doi: 10.1371/ journal.pone.0204011. eCollection 2018.	30235257	S	Rev2/Rev3
Vavla M ( Vavla *et al.*, 2018)	Functional and Structural Brain Damage in Friedreich's Ataxia.	Front Neurol. 2018 Sep 6;9:747. doi: 10.3389/ fneur.2018.00747. eCollection 2018.	30237783	S, F, D	Rev2/Rev3
Mann C ( Mann *et al.*, 2018)	The effect of age on vertex-based measures of the grey- white matter tissue contrast in autism spectrum disorder.	Mol Autism. 2018 Oct 1;9:49. doi: 10.1186/ s13229-018-0232- 6. eCollection 2018.	30302187	S	Rev3/Rev1

### Survey results

**Figure 3.**
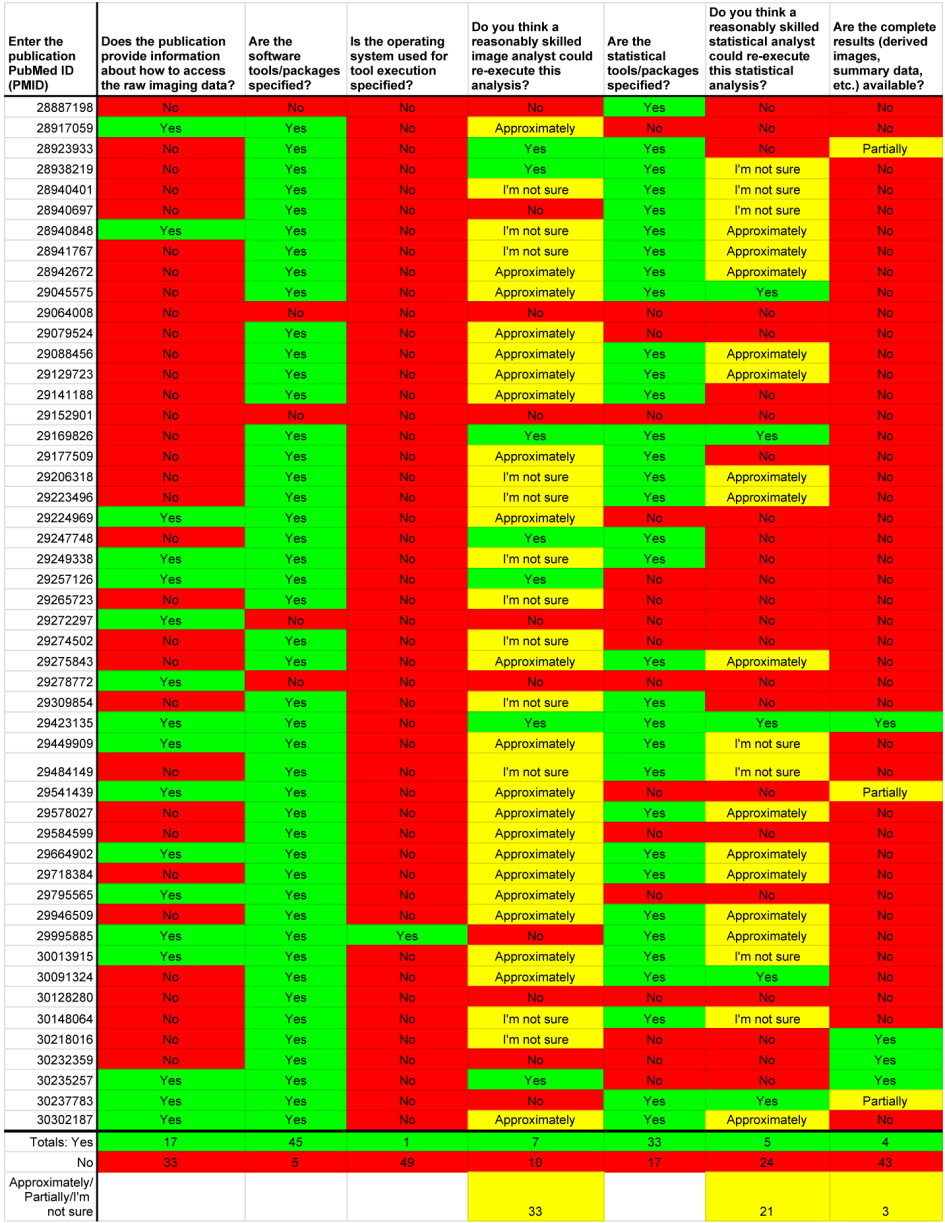
Survey results summary. The 50 publications are summarized on the main factors of data availability, software specification, statistical specification and results availability.

A high-level summary of the survey results are represented in Figure 3. The complete set of question-by-question results are provided in S3 (see *Underlying data*; ( Hodge *et al.*, 2020c).

***Publication availability:*** 38 of the 50 (76%) publications appear to have ‘free full text’ available, according to the PubMed search. Of these, 33 are indexed in PubMed Central. Overall, 43 were freely available through either PubMed Central, Google Scholar or publisher or other websites.

***Data availability:*** 17 of the 50 (34%) publications make reference to the availability of the data used in the publication. However, the publications that indicate availability are mostly reusing data from the large repositories, whereas the publications that do not indicate data availability are principally locally conducted studies. Thus, this indicates that a large fraction of the data being used in publications are not available to the community. 3 of these 17 indicate ‘available upon request’. For the data that is available, the following resources are indicated: ABIDE 1 ( Di Martino *et al.*, 2014), ABIDE 2 ( Di Martino *et al.*, 2017), FCP/INDI ( Mennes *et al.*, 2013), COINS ( Scott *et al.*, 2011), LORIS ( Das *et al.*, 2012), NITRC ( Kennedy *et al.*, 2016), Preprocessed Connectomes Project ( Puccio *et al.*, 2016), UKBiobank ( Miller *et al.*, 2016), Brain Genomics Superstruct Project ( Holmes *et al.*, 2015), ADHD-200 ( HD-200 Consortium, 2012), and Human Connectome Project ( Glasser *et al.*, 2016).

***Image analysis:*** Virtually all of the publications surveyed indicate the imaging analysis software used (45 of 50, 90%). Most publications indicate the use of multiple tools. However, specific tool versions are indicated only about half of the time. Thirty-five different publicly released tools (plus a number of in-house packages) are used in this collection of 50 papers. Not surprisingly, the following tools are used in over 10 publications each: SPM ( Ashburner *et al.*, 1998), FSL ( Jenkinson *et al.*, 2012), and FreeSurfer ( Makris *et al.*, 2003). The specific operating system used is rarely reported (1 of 50, 2%). Overall, our raters felt that in 80% of the publications a skilled image analyst could (or might be able to) repeat the analysis.

***Statistical analysis:*** In approximately two thirds of the publications (66%), the statistical software is indicated, again with variable indication of version and no reporting of the operating system upon which the software was running. In summary, our raters felt that in 29 of the 50 papers (58%), a skilled statistical analyst could (or might be able to) repeat the analysis.

***Results availability:*** Availability of the detailed results is fairly rare. All or partial results are available in seven of the 50 publications (14%). 

***Other observations:*** Two publications which were clinical trials indicated preregistration (with the EU Clinical Trials Register and ClinnicalTrials.gov). None of the non-clinical trials publications reviewed indicated pre-registration ( Nosek *et al.*, 2019).

## Discussion

The recent past literature of autism neuroimaging presents a somewhat consistent picture with respect to the prospects of re-executability with regard to the characteristics we examined in this report. Concerns of this sort have been raised in numerous contexts. The Organization for Human Brain Mapping’s Committee on Best Practices in Data Analysis and Sharing (COBIDAS) ^4^, for example, digs very deeply into the recommendations for reporting and sharing in the literature. The work here is complementary as it takes a high-level gestalt view of re-executability.

Data availability is low, as we would expect to see given the current state of affairs. Figure 3 indicates that there may be a trend towards better data availability (more “Yes” values in the data access column as PubMed ID increases, a good proxy for relative date of publication).

While 80% of the publications were deemed to have repeatable image analysis, the low rate of specifying software version and vanishing rate of specifying operating system is troublesome, since these details can make a difference in results ( Ghosh *et al.*, 2017; Glatard *et al.*, 2015). Even if there are currently only limited software options in some analysis domains, which may implicitly implicate the operating system used, such limitations are not guaranteed to persist through time and should not be assumed for the reader.

A smaller fraction of papers indicates statistical software other than image analysis software, perhaps in the belief that the statistical techniques are more important than the software used to implement the technique.

In both cases there is a distinct difference between the theoretical and practical ability to reproduce both the image analysis and statistical analysis. Rater confidence in the ability to re-execute image analysis and statistical analysis are similar, regardless of the fraction of cases where the software is specified.

The complete results availability criterion was rarely met. Lack of results availability causes a number of problems. Primarily, it is harder to confirm replication (or the degree to which replication was or was not achieved) without the complete set of reported observations, not just the summary tables or figures. Resorting to visual interpretations of ‘similarity’ of published figures remains fraught with issues that can hamper true understanding of new results compared to prior results. Lack of detailed results sharing also compromises subsequent meta-analytic studies that would strive to integrate observations across multiple publications. Finally, lack of complete results exacerbates the publication bias ( Jennings & Van Horn, 2012) through focus on the (relatively few) statistically significant observations while not reporting the large set of observations that are not significant. Examples of complete results availability include when the individual statistical maps for a fMRI analysis are available in a resource such as NeuroVault ^5^, the individual segmentation results of a processing workflow are available at NITRC ^6^ or Zenodo ^7^, etc.

None of the reviewed publications indicated pre-registration ( Nosek *et al.*, 2019). This is not surprising as pre-registration is a fairly new phenomenon, and its uptake in the literature can be expected to take a while. However, as a ‘baseline’ observation, it is still important to note, so that changes in the prevalence of the pre-registration practice can be monitored.

### Limitations

The scope of our survey was rather limited; only 50 publications, and in a selected topic area, autism. However, as a retrospective starting point for evaluation, we believe that it fairly represents the qualitative impressions that investigators have about the nature of neuroimaging publications. We covered numerous neuroimaging subdomains: structural, diffusion, functional; and data and analytic practices in these subdomains can be rather variable. We acknowledge that the details of precise description and dissemination of data and methods may indeed vary by discipline. However, we argue that the ‘best practice’ principles that we are suggesting here are universal and domain-specific solutions are currently available. Also, even though fifty publications are included in the survey, a number of these publications share co-authors or originate from the same research groups. Specifically, 15 of these authors are listed on two or more publications, and 14 of the publications have authors that are also authors on other publications in this set.

The raters (DNK, CH, SMH) we used had over 15 years of neuroimaging research experience each; however, the specialties of each varied from more methodological/statistical to image analytic. This ‘background’ can influence the interpretation of how successfully other ‘reasonably skilled’ investigators could re-execute a given analysis. Familiarity with particular methods can both increase perceived confidence with its reuse (“Of course, everyone knows how to execute that common method”) or decrease confidence (“There are so many details that I know could be varied, how do I know what was really done?”). In the absence of inclusion of explicitly re-executable data and methods in a publication (as in, for example, Ghosh *et al.*, 2017) the interpretation of the precision and completeness of the description with regard to re-executability will be somewhat imprecise and reader-dependent.

Finally, the assessment of each publication is performed on the accessible manuscript as published. It is possible that data and results sharing can have occurred after publication, but this fact may not be represented in the materials reviewed. Indeed, it would be a valuable service to facilitate a more prospective management of these critical re-execution factors that can support authors in making additional supporting data and methods available post publication.

## Conclusions

In conclusion, we feel that the survey results presented here reflect a state of neuroimaging publication practices that leaves ample room for improvement. While reuse of existing data is good, the majority of new data being collected for use in publications is not made publicly available. While the listing of software used is good, important details for reproducibility, such as version, detailed parameters, operating system, etc. are not fully disclosed. Similarly, statistical assessment details are variably reported, making re-execution problematic and approximate. Finally, as very little of the complete results of a publication are disclosed, assessment of the similarity of future replication attempts is severely hampered. Given the overall state of uncertainty about how reproducible (and representative) specific neuroimaging findings are, it seems prudent to begin to tighten up the variables that we as authors do have in order to better support the effective accumulation of knowledge about conditions we study. Promoting best practices in ethical data sharing, complete analytic approach disclosure, and complete results reporting seem to be critical in integrating the complex set of observations we collectively have published about the brain and how it develops and ages. The implications of these observations are that authors should redouble their efforts to be comprehensive in their reporting, even after the publication, to make as accessible as possible the detailed methods and results that they are reporting on. Specifically, authors, reviewers and editors should insist on the complete declaration of: data source and availability status, all software and versions used for data analysis and statistical assessment, the operating system (and version) for data and statistical analysis, and the disposition of the analytic results. Such a ‘checklist’ would be a valuable asset for the community and will be the subject of future efforts. This future checklist should be developed in conjunction with journal specific guidelines, and other checklists (established in conjunction with the COBIDAS report ( Nichols *et al.*, 2017), statistical reporting ( Dexter & Shafer (2017), Nature Neuroscience Reporting Checklist, etc.). In such a way, publishers, editors and reviewers can impart more influence in the manuscripts that they encounter, in an effort to increase the transparency and completeness of the published record that they are playing their role in creating. Together, we hope that we can move the field forward and generate a literature that is more amenable to supporting the understanding of how our collective observations fit together in supporting the understanding of the brain.

## Data availability

### Underlying data

NITRC: CANDI Neuroimaging Access Point: S2_Raw_pubmed_Query_result.csv. http://doi.org/10.25790/bml0cm.68 ( Hodge *et al.*, 2020a)

This project contains the following underlying data:

- S2_Raw_pubmed_Query_result.csv (complete PubMed query result from 1/23/2019)

NITRC: CANDI Neuroimaging Access Point: S3_CompleteSurveyData_v2.xlsx. http://doi.org/10.25790/bml0cm.81 ( Hodge *et al.*, 2020b)

This project contains the following underlying data:

- S3_CompleteSurveyData.xlsx (complete survey results)

### Extended data

NITRC: CANDI Neuroimaging Access Point: S1_Prospects for Reproducibility Check List_V2 - Google Forms.pdf http://doi.org/10.25790/bml0cm.66 ( Hodge *et al.*, 2020c)

This project contains the following extended data:

- S1_Prospects for Reproducibility Check List_V2 - Google Forms.pdf (complete survey form)

Data are available under the terms of the Creative Commons Attribution 4.0 International license (CC-BY 4.0).

## Footnotes

^1^Although maybe not ‘everyone’, depending on the specific details of the data use agreement.

^2^Currently archived in the BrainHack Mattermost ‘general’ channel: https://mattermost.brainhack.org/brainhack/channels/general

^3^A number of publications included data from multiple MRI types.

^4^ http://www.humanbrainmapping.org/cobidas/

^5^ https://neurovault.org/

^6^ https://nitrc.org

^7^ https://zenodo.org/
