# Supplementary material for: An assessment of the autism neuroimaging literature for the prospects of re-executability
Source: F1000Res. 2021 Mar 4;9:1031. Originally published 2020 Aug 24. [Version 2] doi: 10.12688/f1000research.25306.2 (PMC7968525; doi:10.12688/f1000research.25306.2)
Supplement: Peer review history

# Peer review: Reviewer response for version 2

**DOI:** 10.5256/f1000research.54296.r80936

**Published:** 2021-03-15

**Authors:** Karsten Specht

**Affiliations:** 1 Department of Biological and Medical Psychology, University of Bergen, Bergen, Norway

It was a pleasure to have discussed this interesting and important paper with the authors. I hope we will soon see more of those critical surveys, which may improve the reliability of neuroimaging studies and how they are conducted and reported.

Is the work clearly and accurately presented and does it cite the current literature?

Yes

If applicable, is the statistical analysis and its interpretation appropriate?

Yes

Are all the source data underlying the results available to ensure full reproducibility?

Yes

Is the study design appropriate and is the work technically sound?

Yes

Are the conclusions drawn adequately supported by the results?

Yes

Are sufficient details of methods and analysis provided to allow replication by others?

Yes

Reviewer Expertise:

Neuroimaging, fMRI, MRS, reliability

I confirm that I have read this submission and believe that I have an appropriate level of expertise to confirm that it is of an acceptable scientific standard.

# Peer review: Reviewer response for version 1

**DOI:** 10.5256/f1000research.27929.r70151

**Published:** 2020-09-22

**Authors:** Adam Thomas, Travis Riddle

**Affiliations:** 1 Data Science and Sharing Team, Intramural Research Program, National Institute of Mental Health, National Institutes of Health, Bethesda, MD, USA

This paper highlights an important concern regarding the quality of the science seen in published literature. We applaud the authors for undertaking this work, and agree with their general conclusions that there are many opportunities for researchers to improve their reporting. However, we feel that it is worth mentioning a few details in the paper that caused us some concern or confusion.

 First, the paper leads with a summary of some of the issues surrounding reproducibility of science. We urge the authors to make note of another concern that is widely overlooked. Too often researchers communicate a finding based on a convenience sample without any statement indicating that the results might not generalize to a sample that more accurately reflects human diversity (e.g. DeJesus, Callanan, Solis & Gelman, 2019 ^1^; Hruschka, Medin, Rogoff & Henrich, 2018 ^2^; Rad, Martingano & Ginges, 2018 ^3^; Henrich, Heine, & Norenzayan, 2010 ^4^). Of course, this paper is about other reasons for reproducibility, but it seems appropriate to mention this, especially in light of the increased attention given to exclusionary social systems in other domains.

 We also had some concern with the concepts of the 'precision of analysis' (methods paragraph 1). This issue in particular seems difficult to assess reliably, and so there might be a higher degree of measurement error for this concept in comparison to the other concepts. We appreciate that the authors allude to this difficulty later in the paper, when they state that more expertise could also lead to higher levels of measurement error, but here we feel that a more explicit note of caution that these variables in particular should be viewed with additional skepticism.

 The description of how the assessment was applied to each paper was difficult to follow ('survey application' pg 4: "one of three raters applied the survey to each of these articles. Each of the final results..."). Does this mean that each paper was evaluated by 1 reviewer? It seems like it would be useful to have more than one person complete the review. This would allow the reader to have a sense of the degree of inter-rater reliability. If the reliability was low, that would lead us to  be a little more credulous with respect to many of the subsequent findings. If there was more than one reviewer per paper, the authors should report some standard inter-rater agreement metrics. If not, an independent assessment by other raters (along with ratings) would be a wonderful addition to the work, if a bit effort-intensive. Adding a column to figure 2 listing which rater assessed which publication would be helpful. This column could be coded for anonymity (Rater 1, Rater 2) if the authors so choose.

 We additionally found the category of 'results availability' to be a little vague. Especially so since it seems as though papers never reached this cutoff. What does it take for a paper to have complete results availability? Some models might have thousands of parameters or more, and some papers might include dozens or hundreds of fitted models. Does this mean that all parameter values would be reported, confidence intervals, model fit statistics, and so forth would be reported?

Is the work clearly and accurately presented and does it cite the current literature?

Yes

If applicable, is the statistical analysis and its interpretation appropriate?

Yes

Are all the source data underlying the results available to ensure full reproducibility?

Yes

Is the study design appropriate and is the work technically sound?

Yes

Are the conclusions drawn adequately supported by the results?

Yes

Are sufficient details of methods and analysis provided to allow replication by others?

Yes

Reviewer Expertise:

Neuroscience, Psychology, Computer Science, Neuroimaging

We confirm that we have read this submission and believe that we have an appropriate level of expertise to confirm that it is of an acceptable scientific standard.

## References

[1] : Generic language in scientific communication. *Proceedings of the National Academy of Sciences*.2019;116(37) : 10.1073/pnas.1817706116 18370-18377 10.1073/pnas.1817706116 PMC674488331451665

[2] : Pressing questions in the study of psychological and behavioral diversity. *Proceedings of the National Academy of Sciences*.2018;115(45) : 10.1073/pnas.1814733115 11366-11368 10.1073/pnas.1814733115 PMC623310130397139

[3] : Toward a psychology ofHomo sapiens : Making psychological science more representative of the human population. *Proceedings of the National Academy of Sciences*.2018;115(45) : 10.1073/pnas.1721165115 11401-11405 10.1073/pnas.1721165115 PMC623308930397114

[4] : The weirdest people in the world?. *Behav Brain Sci*.2010;33(2-3) : 10.1017/S0140525X0999152X 61-83; discussion 83 10.1017/S0140525X0999152X 20550733

# Response

**Published:** 2021-01-27

**Authors:** David Kennedy

**Affiliations:** University of Massachusetts Medical School, USA

We would like to thank the reviewer for their thoughtful comments and attention to this manuscript. Here we outline our responses to the comments, and indicate where in the manuscript we have made changes.

- We urge the authors to make note of another concern that is widely overlooked. Too often researchers communicate a finding based on a convenience sample without any statement indicating that the results might not generalize to a sample that more accurately reflects human diversity (e.g. DeJesus, Callanan, Solis & Gelman, 20191; Hruschka, Medin, Rogoff & Henrich, 20182; Rad, Martingano & Ginges, 20183; Henrich, Heine, & Norenzayan, 20104). Of course, this paper is about other reasons for reproducibility, but it seems appropriate to mention this, especially in light of the increased attention given to exclusionary social systems in other domains.

**Response: **As we discuss in response to Reviewer 1 above, the details of the subject pool ascertainment and its’ generalizability is beyond the scope of this manuscript, but as this is an important point, we have included it in our updated introduction.

 “In this paper we concentrate on assessing the technical prospects of re-executability of a publication. As introduced above, there are many other factors that will contribute to the actual generalization of the findings including subject population details, data acquisition details, the nature of the processing and statistics (even if they can be re-executed), the underlying biological effect size, if present, etc. (see Figure 1). Take for example, the subject population. Too often researchers communicate a finding based on a convenience sample without any statement indicating that the results might not generalize to a sample that more accurately reflects human diversity (e.g. DeJesus, Callanan, Solis & Gelman, 20191; Hruschka, Medin, Rogoff & Henrich, 20182; Rad, Martingano & Ginges, 20183; Henrich, Heine, & Norenzayan, 20104). Comprehensive and standardized description of all these additional factors are critical as well, but are beyond the scope of this evaluation. Our groups and others are looking into reporting standards for these areas as well.”

  

- We also had some concern with the concepts of the 'precision of analysis' (methods paragraph 1). This issue in particular seems difficult to assess reliably, and so there might be a higher degree of measurement error for this concept in comparison to the other concepts. We appreciate that the authors allude to this difficulty later in the paper, when they state that more expertise could also lead to higher levels of measurement error, but here we feel that a more explicit note of caution that these variables in particular should be viewed with additional skepticism.

**Response: **In order to help the reader appreciate the cautionary note regarding these assessments, we have updated the notion of ‘precision’ to “perceived completeness” to help remind that the precision assessment is in the mind of the assessor.  This is reflected in Methods paragraph one and elaborated upon a little more in Limitations paragraph two:

 “In the absence of inclusion of explicitly re-executable data and methods in a publication (as in, for example, Ghosh, et al.) the interpretation of the precision and completeness of the description with regard to re-executability will be somewhat imprecise and reader-dependent.”

  

- The description of how the assessment was applied to each paper was difficult to follow ('survey application' pg 4: "one of three raters applied the survey to each of these articles. Each of the final results..."). Does this mean that each paper was evaluated by 1 reviewer? It seems like it would be useful to have more than one person complete the review. This would allow the reader to have a sense of the degree of inter-rater reliability.

**Response:** We have attempted to clarify the text regarding the validation cases (pilot assessment) and the dual raters for each publication.

 “The survey was applied to each paper by one of three raters (DNK, SMH, CH). Each of the final results were reviewed by a second rater (DNK or SMH) and consensus reached with the original rater if discrepancies were found.”

  

- [related] an independent assessment by other raters (along with ratings) would be a wonderful addition to the work, if a bit effort-intensive. Adding a column to figure 2 listing which rater assessed which publication would be helpful. This column could be coded for anonymity (Rater 1, Rater 2) if the authors so choose.

**Response:** Table 1 now has a column indicating which raters (Rev1, Rev2 or Rev3) reviewed each publication as the ‘primary’ or ‘checking’ reviewer. 

  

- We additionally found the category of 'results availability' to be a little vague. Especially so since it seems as though papers never reached this cutoff. What does it take for a paper to have complete results availability?

**Response:** We agree that the ‘complete results availability’ was a lofty and somewhat variable goal statement. We have tried to clarify the meaning and ways that this can be satisfied in the updated text of paragraph five in the Discussion:

 “The complete results availability criterion was rarely met. Lack of results availability causes a number of problems. Primarily, it is harder to confirm replication (or the degree to which replication was or was not achieved) without the complete set of reported observations, not just the summary tables or figures. Resorting to visual interpretations of ‘similarity’ of published figures remains fraught with issues that can hamper true understanding of new results compared to prior results. Lack of detailed results sharing also compromises subsequent meta-analytic studies that would strive to integrate observations across multiple publications. Finally, lack of complete results exacerbates the publication bias (Jennings and Van Horn 2012) through focus on the (relatively few) statistically significant observations while not reporting the large set of observations that are not significant. Examples of complete results availability include when the individual statistical maps for a fMRI annalysis are available in a resource such as NeuroVault, the individual segmentation results of a processing workflow are available at NITRC  or Zenodo, etc.”

# Peer review: Reviewer response for version 1

**DOI:** 10.5256/f1000research.27929.r70168

**Published:** 2020-09-22

**Authors:** Karsten Specht

**Affiliations:** 1 Department of Biological and Medical Psychology, University of Bergen, Bergen, Norway

**Summary**

 The article by Hodge and co-workers summarises an attempt in assessing the possibility to replicate 50 published neuroimaging studies on autism. The results indicate that the majority of the studies provide only partial information that would be required for replication of the study. 

 In particular, are information about the operating system missing, only a few studies share their data or other files, and the description of the different analysis steps are sparsely described.

   

**Assessment:**

 The article is well written with a clearly described method and results.

 The study provides a suitable method that could easily be applied to other research topics, as well.

 However, the conclusions that can be drawn from this study are still limited in my view, since it would have been good to include further information in the survey, which I will list below: 

1. In my opinion, the authors focus too much on the technical aspects of a study. Although the authors introduce that a “spectrum-diagnosis” might generate further problems, they do not follow this up in the survey. I would like to see at least one additional column that codes whether the diagnostic criteria and sample are replicable, i.e. are the patients well characterised (age, gender, education), are the diagnostic instrument mentioned, cut-off criteria, etc. 
2. I suggest including another column (at least) in the supplementary material S3 that also lists the imaging modality, i.e. structural MRI, fMRI, MRS, DTI, since they also partly represent different disciplines and traditions in publishing. Further, some methods have only a very limited number of software tools, like MRS, which are often restricted to only one (type of) OS. So, reporting the software may make it almost obsolete to report the OS. 
  Therefore, doing a survey across different neuroimaging modalities may show some general deficiencies, but the other disciplines may need to improve on different aspects.
3. Similarly, concerning fMRI, it also makes a difference whether studies were analysed as whole-brain studies or as a focused region of interest analyses, and, in the latter case, whether the regions were derived from anatomical images or, for example, simply spheres. It would also be informative to know whether studies applied corrected p-values, and which one, and whether the effect sizes were reported.
4. Did the authors control how many studies came from the same lab? Some labs might have a kind of “tradition” in reporting results, which could bias the survey.
5. I think, the headlines of the article are a bit off since the “Discussion” mostly reports the results, and the “Conclusion” primarily discusses the results.

Is the work clearly and accurately presented and does it cite the current literature?

Yes

If applicable, is the statistical analysis and its interpretation appropriate?

Yes

Are all the source data underlying the results available to ensure full reproducibility?

Yes

Is the study design appropriate and is the work technically sound?

Yes

Are the conclusions drawn adequately supported by the results?

Yes

Are sufficient details of methods and analysis provided to allow replication by others?

Yes

Reviewer Expertise:

Neuroimaging, fMRI, MRS, reliability

I confirm that I have read this submission and believe that I have an appropriate level of expertise to confirm that it is of an acceptable scientific standard, however I have significant reservations, as outlined above.

# Response

**Published:** 2021-01-27

**Authors:** David Kennedy

**Affiliations:** University of Massachusetts Medical School, USA

We would like to thank the reviewer for their thoughtful comments and attention to this manuscript. Here we outline our responses to the comments, and indicate where in the manuscript we have made changes.

  

- [...] the authors focus too much on the technical aspects of a study. Although the authors introduce that a “spectrum-diagnosis” might generate further problems, they do not follow this up in the survey. I would like to see at least one additional column that codes whether the diagnostic criteria and sample are replicable, i.e. are the patients well characterised (age, gender, education), are the diagnostic instrument mentioned, cut-off criteria, etc.

**Response:** The survey is indeed focused on the technical ability to reproduce the analytic approach of a study.  We try to do a better job of setting up the scope of the survey in the introduction.  Specifically, we clarify that there are at least three domains in a publication where sufficient information for re-execution needs to be considered: the subject selection (can another researcher generate a comparable group); the data acquisition (can another researcher collect the same data); and the analysis (can another researcher perform the same analysis). All of these areas are important but we are only addressing the ‘analysis’ aspect in this manuscript. Development and application of a similar re-ascertainment survey for the subject selection is an excellent idea, and we hope to pursue such an endeavour in a future survey that would look at subject characteristics such as age, gender, education, diagnostic instruments, etc. 

 We have augmented the second paragraph of the Introduction to address this (and a similar concern raised by Reviewer #2):

 “In this paper we concentrate on assessing the technical prospects of re-executability of a publication. As introduced above, there are many other factors that will contribute to the actual generalization of the findings including subject population details, data acquisition details, the nature of the processing and statistics (even if they can be re-executed), the underlying biological effect size, if present, etc. (see Figure 1). Take for example, the subject population. Too often researchers communicate a finding based on a convenience sample without any statement indicating that the results might not generalize to a sample that more accurately reflects human diversity (e.g. DeJesus, Callanan, Solis & Gelman, 20191; Hruschka, Medin, Rogoff & Henrich, 20182; Rad, Martingano & Ginges, 20183; Henrich, Heine, & Norenzayan, 20104). Comprehensive and standardized description of all these additional factors are critical as well, but are beyond the scope of this evaluation. Our groups and others are looking into reporting standards for these areas as well.”

  

- I suggest including another column (at least) in the supplementary material S3 that also lists the imaging modality, i.e. structural MRI, fMRI, MRS, DTI, since they also partly represent different disciplines and traditions in publishing.

**Response:** We have added a new column to Table 1 that indicates modality. While the details of the data and analysis procedures will vary by these modalities, the need to fully express the complete analysis should be independent of the specific modality.

  

- some methods have only a very limited number of software tools, like MRS, which are often restricted to only one (type of) OS. So, reporting the software may make it almost obsolete to report the OS.

**Response:** While this is certainly true in some situations, we suggest that a good best practice for reporting should be universal (and OS versions change and thus should be disclosed). We have added in the Discussion, third paragraph:

 “Even if there are currently only limited software options in some analysis domains, which may implicitly implicate the operating system used, such limitations are not guaranteed to persist through time and should not be assumed for the reader.“

  

- doing a survey across different neuroimaging modalities may show some general deficiencies, but the other disciplines may need to improve on different aspects.

**Response:** Again, while this is true, the general best practices and principles we’re trying to elucidate here should be universal. What specific disciplines need to do to support these necessary practices may indeed vary by discipline. We try to elaborate on this in the Limitations section, first paragraph:

 “We acknowledge that the details of precise description and dissemination of data and methods may indeed vary by discipline. However, we  argue that the ‘best practice’ principles that we are suggesting here are universal and domain-specific solutions are currently available.”

  

- concerning fMRI, it also makes a difference whether studies were analysed as whole-brain studies or as a focused region of interest analyses, and, in the latter case, whether the regions were derived from anatomical images or, for example, simply spheres.

**Response: **This specific factor is accounted for in the assessors interpretation of how confident they are about re-executing the procedure. If a focused region of interest study is reported, the assessor will determine how confident they are that they could arrive at the ROIs used. 

  

- It would also be informative to know whether studies applied corrected p-values, and which one, and whether the effect sizes were reported.

**Response:** This is an important distinction that we did not clarify in the original manuscript. Our assessment is aimed at evaluating the quality of the reporting (can I do what was reported), rather than the content (is what was reported the right or best thing to do?). The latter assessment is really the purview of the original reviewers of the article itself, whereas the former (in other words, an attempt to generalize a reported finding) is a function that the community of readers would be engaged in and hence our assessment of the feasibility of this from the article.  We’ve added a statement at end of the Survey Design section:

 “Note that our assessments are not if the analysis or data accessibility is ‘optimum’, or even ‘correct’, but rather if the assessor could redo the approach as described.”

  

- Did the authors control how many studies came from the same lab? Some labs might have a kind of “tradition” in reporting results, which could bias the survey.

**Response**: This is a fair point. We have reviewed the author lists of the articles included in the survey and indeed discovered that a number of these articles come from the same groups. This is now explicitly documented in the first paragraph of the Limitations section.

 “Also, even though fifty publications are included in the survey, a number of these publications share co-authors or originate from the same research groups. Specifically, 15 of these authors are listed on two or more publications, and 14 of the publications have authors that are also authors on other publications in this set.”

- the headlines of the article are a bit off since the “Discussion” mostly reports the results, and the “Conclusion” primarily discusses the results.

**Response:** The Results, Discussion and Conclusions sections have been updated to better reflect the appropriate content.
